# Epigenetic Regulation in Neurodegeneration Disease

**DOI:** 10.3390/ijms23116185

**Published:** 2022-05-31

**Authors:** László Bodai

**Affiliations:** Department of Biochemistry and Molecular Biology, Faculty of Science and Informatics, University of Szeged, Közép Fasor 52, H-6726 Szeged, Hungary; bodai@bio.u-szeged.hu

Due to their often age-dependent nature, neurodegenerative diseases impact an increasing portion of modern societies. Finding cures or effective treatments for these disorders has proved to be a rocky road and, most often than not, remains unresolved. We organized this Special Issue in the hope that a better understanding of the roles that chromatin structure alterations and epigenetic dysregulation of transcription play in neurodegeneration may lead to the development of new diagnostic or treatment options. Chromatin is the functional form of the genome that can be modified via specific enzymatic mechanisms to adjust genome function to different cellular needs and environmental stimuli. The main mechanisms of this epigenetic regulation are DNA methylation, nucleosome remodeling, histone post-translational modifications, utilization of histone variants, and RNA-induced transcriptional silencing. Proper epigenetic regulation is essential for appropriate transcriptional activity and its disturbance can lead to or is part of various pathogenic processes. It has been known for decades that chromatin modifications are constituents of the molecular pathomechanisms of several neurodegenerative disorders [1,2], but there is still much to be learned. In this Special Issue, we present three original research articles and two reviews to advance our knowledge in this field.

Histone deacetylase (HDAC) activity is one of the most thoroughly characterized epigenetic factors capable of modulating neurodegeneration. HDAC enzymes can remove acetyl groups from histones and other proteins, and their activity is associated with transcriptional repression. In this Special Issue, Bankole et al. investigated the combined effects of the HDAC class I inhibitor valproate and the SIRT1 activator resveratrol in a murine model of amyotrophic lateral sclerosis (ALS) [3]. Their results show that a combined treatment with valproate and resveratrol delayed disease onset and the decline of motor performance, and improved the lifespan of ALS mice. On the tissue level, the treatment led to increased numbers of surviving motoneurons and decreased microglial activation in the lumbar spinal cord during the end-stage of the disease. Investigating changes in molecular components that might be linked with these phenomena, the authors found reduced overall acetylation of the histone H3 and NF-κB p65 subunit (RelA), but increased Lys130-specific acetylation of RelA in the lumbar spinal cord of ALS mice that were restored to wild-type levels in response to the combined treatment.

In the contribution by Frankowski et al. [4], the authors investigated the effects of loss of histone deacetylase 2 (HDAC2), which was previously implicated in several neurodegenerative disorders [5,6] in a human induced pluripotent stem cell (hiPSC) model. They show that the protein levels of HDAC2 decrease in hiPSCs as they differentiate from neural progenitor cells towards neurons. In hIPSC-derived neurons, knockdown of histone deacetylase 2 (HDAC2) led to increased resistance to cytotoxic agents and a reduced amount of secreted amyloid-beta peptides. These positive effects might be functionally related to concurring changes in transcription and mitochondrial dynamics, as the knockdown of HDAC2 in differentiated neurons led to enhanced mitochondrial respiration and increased mitochondrial length, and altered expression of neural transcription factor TBR1, several synaptic genes, and genes regulating mitochondrial fission and fusion.

Changes in chromatin structure can be also involved in the pathogenesis of genetically determined neurodevelopmental disorders. In a paper by Puente-Bedia et al. [7], the authors described significant structural and functional changes in a mouse genetic model of Down syndrome (DS). They found increased heterochromatinization (an increased amount of the trimethylated-H4K20 epigenetic mark containing heterochromatin) and reduced levels of acetylated histone H4 and global transcriptional activity in hippocampal granular cells of DS mice. These changes were accompanied by changes in the nuclear structure, including a reduced nuclear size, reduced fusion of nucleoli, and decreased number of Cajal bodies.

In this issue, Basavarajappa and Subbanna [8] review the current body of knowledge about histone methylation in several neurodegenerative disorders including Alzheimer’s disease (AD), Parkinson’s disease (PD), Huntington’s disease (HD), and ALS. Histone methylation marks are established by histone methyltransferase or protein arginine methyltransferase enzymes on lysine or arginine residues, respectively, and can be erased by histone demethylases. Different methylation marks might promote different functional outcomes; for example, trimethylation of the H3K4 residue is associated with active promoters, whereas that of H3K27 maintains transcriptional repression. The findings detailed in this review show that both activating and repressing histone methylation marks, most prominently the methylation of H3K4, H3K9, and H3K27 residues, are affected in these disorders and might contribute to transcriptional abnormalities. Importantly, the reviewed data show that pharmacological inhibition of certain methyltransferase or demethylase enzymes improves pathology in disease models.

Finally, in their review article, Zsindely et al. [9] summarized our current knowledge about the role of DNA methylation in Huntington’s disease, a monogenic inherited disease. Methylation of cytosine bases, resulting in 5-methyl-cytosine (5mC), is the most common modification of DNA in mammalian genomes and, also, the most stable epigenetic mark that can be maintained through cell divisions and even inherited to the next generation. In this review, the authors detail mutant Huntingtin-induced changes in DNA methylation, and its effects on transcription, epigenetic age, and genome maintenance, among others. The authors discuss the potential of DNA methylation as a biomarker and also as a potential therapeutic target in HD based on published data demonstrating that the DNA methyltransferase inhibitors 5-aza-2′-deoxycytidine and 5-fluoro-2′-deoxycytidine suppress the mutant Huntingtin-induced pathology in certain models of HD.

We hope that by advancing our understanding of the molecular background of these diseases, the data presented in this Special Issue may contribute to the development of successful diagnostic or treatment strategies for these devastating disorders.
